# Asymptomatic neurocognitive disorders in patients infected by HIV: fact or fiction?

**DOI:** 10.1186/1741-7015-9-138

**Published:** 2011-12-28

**Authors:** Carlo Torti, Emanuele Focà, Bruno M Cesana, Francois X Lescure

**Affiliations:** 1 Institute for Infectious and Tropical Diseases, University of Brescia, P.le Spedali Civili, 125123 Brescia, Italy; 2 Medical Statistics and Biometry Section, University of Brescia, Centro Didattico Polifunzionale, Viale Europa, 11 25123 Brescia, Italy; 3 Infectious and Tropical Diseases Department, APHP, Tenon Hospital, Paris 6 University, Rue de la Chine, 4 75020 Paris, France

**Keywords:** HIV, asymptomatic neurocognitive impairment, HIV dementia, HAART

## Abstract

Neurocognitive disorders are emerging as a possible complication in patients infected with HIV. Even if asymptomatic, neurocognitive abnormalities are frequently detected using a battery of tests. This supported the creation of asymptomatic neurocognitive impairment (ANI) as a new entity. In a recent article published in BMC Infectious Diseases, Magnus Gisslén and colleagues applied a statistical approach, concluding that there is an overestimation of the actual problem. In fact, about 20% of patients are classified as neurocognitively impaired without a clear impact on daily activities. In the present commentary, we discuss the clinical implications of their findings. Although a cautious approach would indicate a stricter follow-up of patients affected by this disorder, it is premature to consider it as a proper disease. Based on a review of the data in the current literature we conclude that it is urgent to conduct more studies to estimate the overall risk of progression of the asymptomatic neurocognitive impairment. Moreover, it is important to understand whether new biomarkers or neuroimaging tools can help to identify better the most at risk population.

Please see related article: http://www.biomedcentral.com/1471-2334/11/356

## Introduction

As HIV-infected patients are living longer thanks to highly active antiretroviral therapy (HAART), our objective as clinicians is to offer them a life expectancy and a quality of life comparable to individuals not infected by HIV. Unfortunately, several complications are emerging as a consequence of premature aging, an effect of persistent or non-reversible immune-activation and concomitant risk factors (such as smoking or use of recreational drugs) or co-infections (such as chronic hepatitis C) [1]. HIV-associated neurocognitive disorders (HAND) could be one of the most important complications, the accepted prevalence of HAND today is approximately 50% despite the overall efficacy of HAART [2]. Indeed, several patients are being diagnosed with a broad spectrum of neurocognitive impairments ranging from subtle alterations that are only evident through specific tests (that is, asymptomatic neurocognitive impairment (ANI)) to mild neurocognitive disorders (MND) that start having deleterious effects on patients' daily activities [3]. Moreover, despite the fact that HIV-associated dementia (HAD) is currently controlled [4], there is a concern that neurocognitive disorders will worsen even on HAART, leading to a resurgence of this problem in the future. This concern is based on patients on HAART who have neurological deficits despite persistent undetectable HIV RNA in plasma [5], but with detectable HIV RNA in the cerebrospinal fluid (CSF) [6], neuroinflammation in the CSF [7], or ß-amyloid deposition in the brain [8].

The American Academy of Neurology (AAN) updated the nosology of HAND in 2007, introducing ANI as a new pathological entity [9]. The panel itself recommended using this classification only for research. Indeed, this entity had been evaluated in only two studies concerning a small number of ANI-suffering patients before consensual but not unanimous approval [10,11]. Notwithstanding this consideration, it has already been adopted into clinical practice. For instance, the Italian guidelines for HIV disease management suggest starting HAART in all patients with HAND, a recommendation that is stronger for those with MND but also applies to those with ANI [12].

In the accompanying paper published in *BMC Infectious Diseases*, Gisslèn *et al. *[13] emphasize that a high proportion of HIV infected patients (about 20%) may be classified as neurocognitively abnormal (though asymptomatic) using the AAN criteria. According to this classification, ANI is characterized by neuropsychological testing outcomes that are at least one standard deviation below the mean of normative scores in at least two cognitive areas among at least five domains. The authors highlighted that, according to this criterion, even in a general population with a normal distribution of the neuropsychological testing outcomes, almost 16% of the individuals will be defined as abnormal. The bottom line is that the definition of ANI may lead to an unacceptable false-positive rate and that therefore the actual problem is overestimated.

## Discussion

### Statistical considerations

From a statistical point of view, in addition to the results of the paper by Gisslèn *et al. *[13], it is interesting to note that in the case of independent tests exhibiting a Gaussian distribution the probability of having all scores of the five tests more than one standard deviation from their mean is only about 0.42 (as demonstrated both by the probability of only one test being abnormal, which is 0.84134, raised to five and by simulations that we have performed); so, the probability of having something not 'absolutely normal', according to the AAN proposed threshold (one standard deviation lower than the mean), is less favorable than tossing a coin. Of course, the very high probability value of making a diagnosis of ANI together with the above result immediately emphasizes the problem of the false positive proportion and, consequently, the need for a critical consideration of the validity of diagnostic criteria based on the 'z-score'. Indeed, it is common to obtain some z-score values associated with a low probability value, meaning that it is practically impossible from a Gaussian distribution [14]. Moreover, the Gaussian model could be limited since it may include impossible negative values; this happens, for instance, for values lower than two standard deviations less than the mean of the raw values (that is, the neuropsychological test results without adjusting for the normative values). According to these considerations, it would be more advisable not to rely upon some asymptotic results from the statistical theory (central limit theorem) leading to the Gaussian model, but to refer to skewed distributions and to calculate non-parametric thresholds (0.95 quantile, for example, or its lower 95% confidence limit). We actually think that, when an immediate medical treatment is not required as in the case of an ANI, knowing that a patient is under some relevant quantiles (0.5, 0.10 or 0.25) of the 'reference population' can be more informative for monitoring his/her follow-up than a result based on a parametric model (one standard deviation less than the mean), which may be too restrictive and not able to fit well with a set of real observations.

### Clinical considerations

From a clinical point of view, it is important to highlight that, being asymptomatic, ANI is not an overt disease, so it would be clinically relevant only if it had been correlated with signs of definite pathologies or if it had been shown to be predictive of more severe neurocognitive impairments. Although a cross-sectional study demonstrated a correlation between pathological evidence of HIV encephalitis at autopsy and ANI diagnosed ante-mortem, this study is limited because it involved only the most compromised patients who died, so results are not transferable to the general population [11]. Moreover, it is a little disconcerting to find that, using the AAN criteria, even 16% to 19% HIV-negative young people about 35 years old who were used as controls had neurocognitive disorders in the study by Heaton *et al. *[3]. Lastly, in the five years after ANI was defined [9], no single study was performed to assess its predictive value for MND or HAD. Therefore, it remains plausible that patients with ANI have a greater risk of progression to MND/HAD than those without, but this is currently uncertain and may be difficult to demonstrate for several reasons:

(i) since neurocognitive impairment has been correlated with a variety of conditions (such as psychiatric disorders or psychoactive medications, recreational drugs, alcohol abuse, chronic hepatitis C, metabolic abnormalities, vascular disease, and even potential neurotoxicity of antiretroviral drugs) [15-22], it is difficult to properly select the cases where the effect of HIV is predominant, which are more probably destined to MND and HAD;

(ii) HAART is likely to improve the evolution of HIV-associated neurocognitive impairment [23], so studies on the natural history of this disease are difficult to conduct;

(iii) neurocognitive impairment may fluctuate over time [24], implying that the disease may spontaneously recover or that the prodromic stages of dementia (ANI and MND) are not strongly predictive. This has been already proven for mild cognitive impairment in aging-associated cognitive decline [25];

(iv) the pathogenesis of neurocognitive disorders involves multiple signs [26], whose relationships are for the most part not elucidated. It is possible that neurocognitive disorders comprise heterogeneous conditions whose prognosis and treatment are different. So the question is: can new biomarkers and/or neuroimaging techniques help disentangle the different nosological entities?

Several studies have investigated biomarkers and found that signs of persistent immune-activation in the CSF (such as pleocytosis, increased albumin concentration, neopterin, neurofilaments, IL-6, cholesterol, myoinositol) [7,27-30] or chronic peripheral activation (indicated by CD14, CD16 or lipopolysaccharide) [31-33] correlated with neurological impairment. As for neuroimaging, recently developed tools are promising. For instance, magnetic resonance spectroscopy is able to show a decreased level of N-acetyl aspartate, a sign of mature neurons and their axonal processes, in the basal ganglia of patients infected by HIV; moreover, decreased levels of N-acetyl aspartate were accompanied by increased choline and myoinositol as signs of cell turnover and inflammation [34]. Interestingly, these markers may be restored by HAART, and this may be correlated with improvement of neurocognition [35]. It has to be seen whether incorporation of these biomarkers or neuroimaging results into diagnostic algorithms may improve the clinical predictive value and relevance of subtle neurocognitive impairments such as ANI.

### Considerations for clinical management

In the meantime, what should clinicians do when a diagnosis of ANI is made? Clearly, with the current diagnostic definition, ANI should not be assumed to be synonymous with an overt pathology or a major risk for HAD. Some experts recommend, as a cautious approach, to retest patients with ANI with the aim of identifying early on, any sign of progression of the neurocognitive disorder [12]. However, this practice should not excessively preoccupy our patients, leading to anxiety and depression for a condition that may not be significant. This is important in light of the fact that some patients, up to 53%, suffer from ANI (as currently defined) [5].

## Conclusions

In principle, the burden of ANI as a possible disease is huge in the HIV infected population. However, at present, this condition is more theoretical than real because it has not been validated for its relevance in clinical practice. Appropriately designed studies are urgently needed to understand whether this ANI is clinically meaningful or a false alarm both for patients and for physicians. In other words, a clinical validation of ANI is a priority before strong clinical recommendations are made. Since this condition may be heterogeneous, both in terms of pathogenesis and outcome, it has to be ascertained whether new biomarkers and neuroimaging techniques can help identify the most at risk patients. In the meantime, a cautious approach is recommended to monitor patients with ANI for progression of their neurological impairment, but this should be done without worrying our patients too much.

## Abbreviations

AAN: American Academy of Neurology; ANI: asymptomatic neurocognitive impairment; CSF: cerebrospinal fluid; HAART: highly active antiretroviral therapy; HAD: HIV-associated dementia; HAND: HIV-associated neurocognitive disorder; IL-6: interleukin-6; MND: mild neurocognitive disorder.

## Competing interests

The authors declare that they have no competing interests.

## Authors' contributions

All authors conceived the concept and design of the commentary. CT, EF and FXL wrote the clinical part of the commentary. BMC wrote the statistical part of the commentary. All authors contributed to revision and approval of the final commentary for submission.

## Pre-publication history

The pre-publication history for this paper can be accessed here:

http://www.biomedcentral.com/1741-7015/9/138/prepub
